# Traumatic Brain Injury Severity, Neuropathophysiology, and Clinical Outcome: Insights from Multimodal Neuroimaging

**DOI:** 10.3389/fneur.2017.00530

**Published:** 2017-10-05

**Authors:** Andrei Irimia, Sheng-Yang Matthew Goh, Adam C. Wade, Kavi Patel, Paul M. Vespa, John D. Van Horn

**Affiliations:** ^1^Ethel Percy Andrus Gerontology Center, USC Leonard Davis School of Gerontology, University of Southern California, Los Angeles, CA, United States; ^2^Laboratory of Neuro Imaging, Mark and Mary Stevens Neuroimaging and Informatics Institute, Keck School of Medicine, University of Southern California, Los Angeles, CA, United States; ^3^Brain Injury Research Center, Department of Neurosurgery, David Geffen School of Medicine, University of California, Los Angeles, Los Angeles, CA, United States

**Keywords:** traumatic brain injury, clinical outcome, Glasgow Coma Scale, Glasgow Outcome Score, post-traumatic seizures, neuroimaging, magnetic resonance imaging

## Abstract

**Background:**

The relationship between the acute clinical presentation of patients with traumatic brain injury (TBI), long-term changes in brain structure prompted by injury and chronic functional outcome is insufficiently understood. In this preliminary study, we investigate how acute Glasgow coma score (GCS) and epileptic seizure occurrence after TBIs are statistically related to functional outcome (as quantified using the Glasgow Outcome Score) and to the extent of cortical thinning observed 6 months after the traumatic event.

**Methods:**

Using multivariate linear regression, the extent to which the acute GCS and epileptic seizure occurrence (predictor variables) correlate with structural brain changes (relative cortical atrophy) was examined in a group of 33 TBI patients. The statistical significance of the correlation between relative cortical atrophy and the Glasgow Outcome Score was also investigated.

**Results:**

A statistically significant correlative relationship between cortical thinning and the predictor variables (acute GCS and seizure occurrence) was identified in the study sample. Regions where the statistical model was found to have highest statistical reliability in predicting both gray matter atrophy and neurological outcome include the frontopolar, middle frontal, postcentral, paracentral, middle temporal, angular, and lingual gyri. In addition, relative atrophy and GOS were also found to be significantly correlated over large portions of the cortex.

**Conclusion:**

This study contributes to our understanding of the relationship between clinical descriptors of acute TBI, the extent of injury-related chronic brain changes and neurological outcome. This is partly because the brain areas where cortical thinning was found to be correlated with GCS and with seizure occurrence are implicated in executive control, sensory function, motor acuity, memory, and language, all of which may be affected by TBI. Thus, our quantification suggests the existence of a statistical relationship between acute clinical presentation, on the one hand, and structural/functional brain features which are particularly susceptible to post-injury degradation, on the other hand.

## Introduction

Long-term clinical outcome after traumatic brain injury (TBI) is predicated upon a large variety of often poorly understood factors which substantially complicate the task of identifying the relationship between acute clinical variables and chronic functional deficits. Nevertheless, understanding how post-TBI cortical atrophy patterns reflect acute-stage patient presentation may help to identify cortical areas that are likely to undergo substantial atrophy, and implicitly to isolate aspects of cognitive, affective and neural function which are at highest risk for long-term degradation.

Attempts to relate TBI-related changes in brain structure to clinical variables often involve structural brain variables provided by neuroimaging methodologies, such as magnetic resonance imaging (MRI) and diffusion tensor imaging (DTI) (1–3). In previous studies, quantitative metrics provided by acute neuroimaging of TBI patients have been used to describe the relationship between acute injury profiles and chronic dysfunction (4–7). By contrast, hardly any non-neuroimaging clinical variables have been identified which can be used to elucidate the pattern of structural brain changes after TBI. Nevertheless, the ability to incorporate such non-neuroimaging clinical descriptors into outcome forecasting models is important because many such descriptors—including the Glasgow Coma Score (GCS)—are recorded routinely by clinicians and relied upon during the treatment decision-making process.

In this study, we illustrate how two important TBI severity indicators that are routinely assessed by clinicians in the acute care setting and without the use of neuroimaging can be used to relate patient presentation in the acute stage of TBI to the pattern and extent of post-TBI cortical atrophy as well as to neurological outcome. These two indicators—the GCS and the occurrence of epileptic seizures during the acute stage of TBI—can likely assist in predicting cortical atrophy patterns and in evaluating the risk for poor neurological outcome. This study additionally identifies cortical regions whose susceptibility to post-traumatic atrophy is correlated significantly and reliably—in a statistical sense—with functional outcome and with clinical descriptors of TBI severity.

## Materials and Methods

### Patients

The study was designed and implemented in accordance with the Declaration of Helsinki, the U.S. Code of Federal Regulations (45 CFR 46), and with the approval of the Institutional Review Boards at the University of California, Los Angeles (UCLA), and the University of Southern California (USC). Signed informed consent was obtained from each patient or from their legally authorized representative prior to the study. A total of *N* = 33 TBI patients admitted to the Neurointensive Care Unit (NICU) at the UCLA Ronald Reagan Medical Center were included in the study (23 males). The mean and SD of the sample were 33.6 and 16.5 years, respectively. The age range was 18–62 years, and 15 patients were younger than the average volunteer age. The skewness and kurtosis, respectively, were 0.61 and 2.34 years, indicating that the sample is (1) moderately skewed toward older adults and (2) platykurtic (i.e., with relatively few patients whose ages were close to either limit of the age range in the sample). A total of 15 patients had acute seizures. The mechanism of injury involved falls (*N* = 9), motor vehicle accidents (*N* = 8), pedestrians hit by motor vehicles (*N* = 8), motorcycle accidents (*N* = 5), bicyclists hit by motor vehicles (*N* = 2), and a gunshot wound victim (*N* = 1). Patients received a loading dose of phenytoin (18 mg/kg) upon hospital admission and continued to receive this medication (300 mg/day) for at least 7 days. Daily trough levels of total serum phenytoin were measured and additional boluses were provided to maintain levels between 10 and 20 mg/dl. If seizures were detected, additional phenytoin was administered to raise levels to a range of 18–25 mg/dl. Phenobarbital was added as a daily anti-epileptic agent if seizures recurred on withdrawal of pentobarbital or propofol.

### Data Acquisition

The general patient management protocol is described elsewhere (8). Briefly, continuous monitoring of EEG waveforms was initiated at the patient’s bedside at the earliest opportunity after NICU admission. EEG traces were continuously displayed at the bedside for on-line observation by clinical staff. A physician with training in the interpretation of EEG readings reviewed EEG activity at least three times per day and additionally when alerted by the bedside nurse of suspicious EEG activity. Upon detection of such activity, clinical staff implemented patient management changes as deemed appropriate.

The Glasgow Coma Score-Extended (GCS-E) was recorded upon NICU admission (mean GCS-E: 6.15 ± 3.36). Pupil sizes upon hospital admission were also recorded. Electroencephalographic (EEG) measurements were monitored continuously at the patient’s bedside starting immediately after admission to NICU. Seizures that occurred within the first week after injury were detected by the NICU nurse or neuro-intensivist in one of three ways: online identification, during EEG screening, or by the total power trend seizure detection method (8). All the seizures recorded and included in this study were partial seizures with secondary generalization.

Structural *T*_1_-weighted MRI volumes were acquired using a 3-T Trio Tim MRI scanner (Siemens AG, Erlangen, Germany) with the following acquisition parameters: repetition time (TR) = 1,900 ms, echo time (TE) = 3.52 ms, flip angle (FA) = 9°, inversion time (TI) = 900 ms, voxel size = 1 mm × 1 mm × 1 mm, phase field of view (FOV) = 100%, sampling = 100%, matrix = 256 × 256. Scanning sessions were held both several days (acute baseline) as well as 6 months (chronic follow-up) after TBI, and the same MRI scanner and acquisition parameters were used in both cases. The average Glasgow Outcome Score-Extended (GOS-E) at 6 months post-injury was 5.43 ± 2.51.

### Image Processing

Data processing workflows were generated using the LONI Pipeline (pipeline.loni.usc.edu), and segmentation and regional parcelation were performed using FreeSurfer (9, 10). For each subject, the cortical surface was reconstructed as a triangular tessellation with an average inter-vertex distance of ~1 mm to produce a high-resolution, smooth representation of the white matter (WM)/gray matter (GM) interface, as detailed elsewhere (11). At each vertex of the tessellation, cortical thickness was measured as the distance between the WM/GM boundary and the cortical surface. A total of 74 cortical structures (gyri and sulci) were identified and parceled using a probabilistic atlas (12). Briefly, neuroanatomical labels were assigned to voxels based on probabilistic information estimated from a manually labeled training set. The method uses the previous probability of a tissue class occurring at a specific atlas location and the probability of the local spatial configuration of labels given each tissue class. The technique is comparable in accuracy with manual labeling (13).

### Statistical Analysis

The statistical analysis involved two separate steps. In the first step, we aimed to determine whether the GCS-E and acute seizure occurrence could predict GM atrophy across the cortex. In the second step, our purpose was to test the hypothesis that GM atrophy could predict the GOS-E at 6 months post-injury.

In Step 1, for each gyrus and sulcus, we sought to identify the extent to which GCS-E and seizure occurrence while in the NICU can be used as predictor variables in a multivariate regression model where the response variable is structural outcome (relative cortical atrophy) at 6 months after injury. In other words, the two predictor variables were included in the design matrix of a linear model whose response variable was the relative difference in cortical thickness between acute and chronic time points. The null hypothesis was that there is no statistically significant correlation between the predictor variables and the response variable. The hypothesis was tested for each gyrus and sulcus following standard multivariate statistical inference theory, as described in detail in Chapter 10 (pp. 337–343, in particular) of the classic textbook *Methods of Multivariate Analysis* by Rencher (14). Null hypotheses were rejected at α = 0.05 subject to corrections for multiple comparisons *via* the false discovery rate (FDR) approach (15). We implemented a random-effects, multiple correlation analysis approach where the design matrix ***X*** contained the acute GCS-E and seizure occurrence score, the latter being coded as a binary variable (0 or 1), for a total of *q* = 2 predictor variables. In other words, the GCS-E and the seizure occurrence score are treated here as random effects, where the term *random* implies that the values of these variables are not under the control of the researcher. The response matrix ***Y*** contained either a structural outcome variable (the relative change in cortical thickness as a function of time) or a functional outcome variable (the GOS-E at 6 months post injury, coded as a categorical variable), resulting in one response variable (*p* = 1). Age at injury and patient sex were regressed out before the main multivariate analysis was implemented. The matrix ***B*** contains the least-squares estimators to the set of multivariate regression equations and is of the form

$$B={\left(X^{T}X\right)}^{-1}X^{T}Y.$$

The hypothesis and error matrices ***H*** and ***E***, respectively, are given by

$$H=Y^{T}Y-B^{T}X^{T}Y$$

and

$$Ε=Y^{T}Y-N({\bar{Y}}^{T}\bar{Y}),$$

where $\overset{¯}{\left(\cdot \right)}$ is the mean over subjects. Let $\nu_{H}=q$ be the degrees of freedom (d.f.) for ***H*** and ν*_E_* = *N* − *q* − 1 be the d.f. for ***E***, each and *p* be the number of response variables. With

$$w=\nu_{E}+\nu_{H}-\frac{1}{2}(p+\nu_{H}+1)$$

and

$$t=\sqrt{\frac{p^{2}\nu_{H}^{2}-4}{p^{2}+\nu_{H}^{2}-5}}$$

the omnibus test statistic is Wilks’ Λ statistic, which can be converted to an *F* statistic with *d*_1_ and *d*_2_ d.f. using the transformation

$$F=\frac{{\left(1-\Lambda \right)}^{1/t}}{\Lambda^{1/t}}\frac{d_{2}}{d_{1}}.$$

The degrees of freedom *d*_1_ and *d*_2_ are given by

$$d_{1}=p\nu_{H},$$

$$d_{2}=wt-\frac{1}{2}(p\nu_{H}-2).$$

Cortical thickness measurements which could not be appropriately made due to loss of image contrast in the presence of lesions were treated as missing data, and mean substitution was used for data imputation to account for missing values. For any given gyrus or sulcus, the null hypothesis was not tested unless cortical thickness measurements were available for at least 90% of subjects. Statistical validity was assessed by randomly permuting design matrix rows 10,000 times and then re-computing the *F* statistic for each permutation. The actual *F* statistic was then compared against the distribution of *F* statistics obtained during the permutation process and the null hypothesis was rejected at α = 0.05 subject to the FDR correction.

In Step 2 of the statistical analysis, the correlation coefficient *r* between relative regional GM atrophy and the GOS-E (both evaluated at 6 months after injury) was computed and its significance was tested using the statistic

$$t=\frac{r}{\sqrt{\left(1-r^{2})/(N-2\right)}},$$

which exhibits a *T* distribution with *N* − 2 d.f.

For both Steps 1 and 2 described above, power analyses were implemented. First, effect sizes were quantified using Cohen’s *f*^2^, defined as

$$f^{2}=\frac{R^{2}}{1-R^{2}},$$

where *R*^2^ is the coefficient of determination. Second, the empirically estimated effect sizes were used to calculate the minimum sample sizes required to test statistical hypotheses at power and significance levels of 0.8 and 0.05, respectively. The power *P* of each statistical test was calculated using a standard formula (16) involving the non-central *F* distribution with non-centrality parameter λ = *nf*^2^.

## Results

For Step 1, the power analysis indicated that our sample size of *N* = 33 was sufficient to test statistical hypotheses at a power level of 0.8 and significance threshold of 0.05 only if the effect size was greater than or equal to 0.33. For step 2, the minimum required effect size was 0.25. The percentage of cortical regions associated with effect sizes below the required thresholds for Steps 1 and 2 of the analysis were 12 and 9%, respectively. None of the hypothesis tests associated with any of these latter regions were found to be significant, nor are any reported in this study.

**Figure 1:**
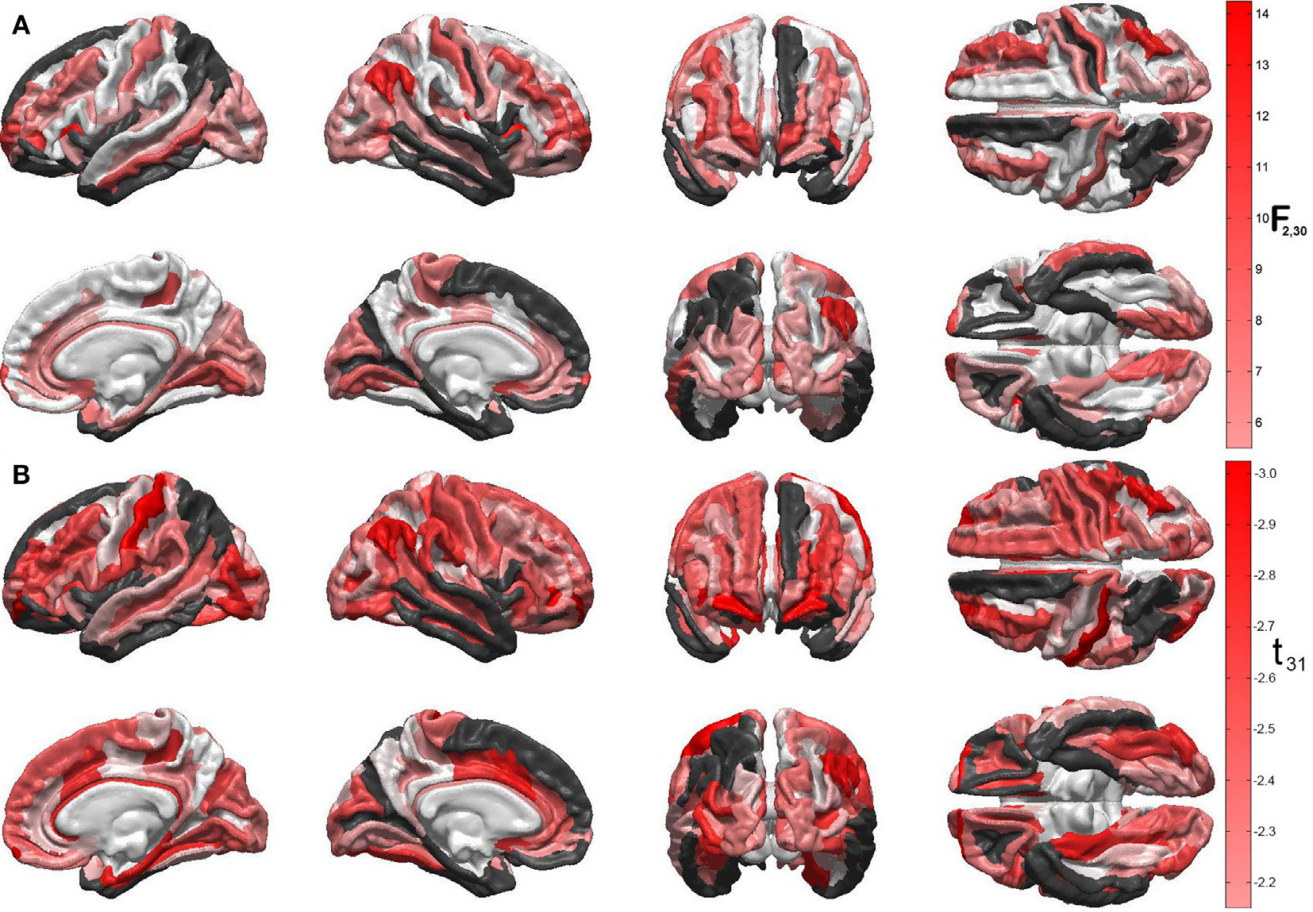
**(A)** Quantification of the linear model’s ability to predict cortical atrophy extent at 6 months after injury. For each gyrus and sulcus, the null hypothesis that there is no statistically significant correlation between the predictor variables and the response variable (cortical thinning, in millimeters) was tested. Values of the *F*_2,30_ statistic for each statistical test are encoded on the cortical surface, subject to the false discovery rate correction for multiple comparisons. Darker red hues indicate higher significance of the statistical test and, consequently, stronger ability to predict cortical thinning for the areas in question. Regions where the null hypothesis was not tested because less than 90% of cortical thickness data were available (see text) are drawn in black. Regions where the test statistic was lower than the threshold *F* statistic of the reliability analysis permutation test are drawn in white. **(B)** Statistical significance of the correlation between relative cortical atrophy and the GOS-E. Values of the *t*_31_ statistic for each statistical test are encoded on the cortical surface, as in panel **(A)**. Note that all values of this statistic are negative, which confirms that greater regional atrophy is associated with lower GOS-E values (i.e., poorer functional outcome), as expected. The values of *F* and *t* statistics in (A) and (B), respectively, are associated with different statistical tests and different degrees of freedom and, therefore, they should not be compared to one another.

Figures 1A,B display the results of Steps 1 and 2 of the statistical analysis, respectively. Specifically, Figure 1A shows the *F* statistics associated with the ability of the two predictor variables (acute GCS and post-traumatic epileptic seizure occurrence) to forecast cortical atrophy across the TBI sample examined. The cortical maps of the *F* statistic—with values summarized in Table 1—indicate that the linear model has high reliability in predicting the value of the response variable in regions which are comparatively large and anatomically prominent, such as the transverse frontopolar gyri (*F*_2,30_ = 11.21, *p* < 0.0002), middle frontal gyri (*F*_2,30_ = 6.19, *p* < 0.0056), postcentral gyri (*F*_2,30_ = 7.84, *p* < 0.0018), angular gyri (*F*_2,30_ = 14.17, *p* < 0.0001), and lingual gyri (*F*_2,30_ = 6.78, *p* < 0.0037). Additional regions for which the null hypothesis is rejected at the α = 0.05 significance level are located on the lateral aspects of the temporal and occipital lobes, as well as in insular cortex; some insular and limbic structures are also to be noted. A clear relationship between ictogenesis locations—as determined based on the locations of scalp EEG electrodes—and the regions which underwent greatest atrophy was not identified, possibly due to the need for anatomically constrained inverse localization of electrical activity to the cortical surface.

**Table 1:** Cortical regions whose 6-months atrophy is significantly correlated with both Glasgow coma score and with the seizure occurrence score (0 or 1).

Lobe	Cortical structure	*F*_2,30_	*p*	*f*^2^	1 − β
Frontal	Transverse frontopolar gyrus/sulcus	11.21	0.0002	0.3557	0.8386
	Frontomarginal gyrus/sulcus	7.13	0.0038	0.4122	0.8907
	Middle frontal gyrus	6.19	0.0056	0.7213	0.9902
Insular	Anterior circular insular sulcus	6.88	0.0043	0.5211	0.9509
	Anterior lateral sulcus, vertical part	7.16	0.0037	0.8267	0.9960
	Superior circular insular sulcus	7.18	0.0028	0.3775	0.8608
	Inferior circular insular sulcus	6.82	0.0044	0.4614	0.9233
	Posterior lateral sulcus	7.85	0.0027	0.4329	0.9057
Limbic	Anterior cingulate gyrus/sulcus	7.20	0.0037	0.4030	0.8834
	Middle anterior cingulate gyrus/sulcus	5.72	0.0079	0.4610	0.9231
	Subcallosal gyrus	7.03	0.0031	0.6416	0.9810
	Cingulate sulcus, marginal part	9.84	0.0010	0.7567	0.9927
	Posterior ventral cingulate gyrus	7.19	0.0037	0.4106	0.8895
Temporal	Anterior transverse collateral sulcus	5.95	0.0070	0.6268	0.9786
	Transverse temporal sulcus	6.41	0.0055	0.7823	0.9941
	Middle temporal gyrus	9.68	0.0011	0.7316	0.9910
Parietal	Postcentral gyrus	7.84	0.0018	0.4771	0.9317
	Paracentral lobule/sulcus	8.84	0.0010	0.6195	0.9773
Occipital	Lingual gyrus	8.28	0.0014	0.3932	0.8751
	Anterior occipital sulcus	9.92	0.0010	0.4115	0.8902
	Inferior occipital gyrus/sulcus	7.17	0.0037	0.4799	0.9331
	Cuneus	6.09	0.0060	0.3567	0.8397
	Occipital pole	6.63	0.0049	0.7921	0.9946

*For structures within each lobe, the test statistic (*F*_2,30_), *p*-value, effect size (*f*^2^), and statistical power (1 − β) of the test are listed. For brevity, regions for which the statistical test is relatively underpowered (i.e., 1 − β < 0.8) and for which the multiple correlation is not statistically significant (i.e., *p* > 0.05, corrected for multiple comparisons) are not displayed*.

Figure 1B shows the *t* statistics associated with the significance of the linear correlation between the regional atrophy in each cortical parcel, on the one hand, and the GOS-E at 6 months post injury, on the other hand. Negative correlation coefficients between the two metrics are observed because, as expected, neurological outcome is poorer (smaller GOS-E) as atrophy is more substantial. The regions with strongest correlations between the two measures are found to be the postcentral gyri (left hemisphere: *r* = −0.47, *t*_31_ = −2.97, *p* < 0.0029; right hemisphere: *r* = −0.40, *t*_31_ = −2.46, *p* < 0.0098), the frontomargial gyri (left hemisphere: *r* = −0.47, *t*_31_ = −2.93, *p* < 0.0032; right hemisphere: *r* = −0.46, *t*_31_ = −2.89, *p* < 0.0035), the angular gyri (right hemisphere only: *r* = −0.43, *t*_31_ = −2.63, *p* < 0.0066), and the middle occipital gyrus (left hemisphere: *r* = −0.41, *t*_31_ = −2.52, *p* < 0.0086; right hemisphere: *r* = −0.37, *t*_31_ = −2.21, *p* < 0.0173). Additional regions of significant correlation between relative atrophy and the GOS-E are located on the dorsolateral aspect of the frontal lobe, lateral and medial aspects of the temporal lobe, as well as in limbic areas.

## Discussion

Our findings contribute to ongoing efforts aimed at determining how acute clinical presentation variables can be combined in the form of a composite score to early predict clinical outcome. The appropriate interpretation of such a score could provide the possibility to relate the nature and severity of cortical thinning in TBI patients to acute clinical variables which modulate brain atrophy and functional decline or recovery. Though independent validation of our findings in larger cohorts is required, potential applications of our findings include (1) guiding rehabilitation efforts based on early prediction of specific brain functions at high risk for degradation, (2) understanding which acute clinical variables can be used to forecast clinical outcome in the context of substantial case heterogeneity in TBI studies, and (3) formulating hypotheses on how acute clinical variables can modulate the pattern and extent of long-term neural and cognitive dysfunction. Because early treatment of TBI sequelae is essential for determining outcome (17), the first of these three potential applications is likely the most critical one, particularly given clinicians’ need to tailor rehabilitation protocols according to the type of specific deficits which individual patients are most likely to develop.

In this study, null hypotheses were not tested unless cortical thickness measurements were available for at least 90% of volunteers. Thickness measurements were unavailable for gyri or sulci whenever these brain regions contained lesions which modified the GM/WM contrast in *T*_1_-weighted MRI to such an extent that the boundary between these tissue types could not be identified unambiguously. The implication of this limitation is that our study does not provide insights into the statistical relationship between GCS, seizure occurrence, and cortical thickness for regions where thickness measurements were unavailable to us in more than three volunteers. Nevertheless, (1) the atrophy of brain regions affected by gross, primary injury has been documented extensively (18–21) and (2) atrophy in such regions has been strongly correlated with GCS [see Ref. (22) and references therein] and with neuropathophysiological manifestations originating in or affecting these regions (23–25). Partly for these reasons, it is conceivable that the statistical relationships identified in this study may also hold for brain structures affected by primary injuries.

In addition to suggesting an important link between the severity of acute brain function disruption and chronic functional outcome, our study confirms and provides interesting details on how regional atrophy can modulate functional impairment in the chronic stage of TBI. For example, frontopolar cortex is known to be involved in executive processing during working memory (26), moral sensitivity (27), response inhibition (28), and emotional regulation (29), which are often affected by TBI. Similarly, the middle frontal gyri are involved in spatial memory (30), attention (31), and inhibitory control (32). The angular and lingual gyri have been shown to be activated during speech (33), reading (34, 35), writing (36), face recognition (37), visual acuity tasks (38), and arithmetic processing (39, 40). Another cortical structure for which the multivariate model holds substantial reliability is the postcentral gyrus, which is the locus of the primary somatosensory cortex (S1). Thus, this study suggests that the clinical variables investigated here may be predictive of cortical atrophy and of neurological outcome in regions which are responsible for important executive functions, and which often degrade following TBI. Understanding the relationship between cortical degradation in brain areas associated with these functions, on the one hand, and bedside clinical measures, on the other hand, may be useful to clinicians, rehabilitation professionals and to social workers as they seek to formulate appropriate rehabilitation protocols that target sensory, linguistic, and analytic processing abilities in TBI survivors.

Our investigation suggests that the spatial pattern of brain atrophy and the severity of neurological outcome may be predictable, to some extent, based on knowledge of acute function (the GCS-E) and of epileptic seizure occurrence. This supports the notion that monitoring epileptiform activity in the acute stage of TBI can be very important for identifying TBI patients who are at risk for poor outcome, and that such monitoring can serve as an important inferential factor in addition to the GOS when attempting to identify patients who can benefit from supervision using EEG and other techniques for measuring the electrical activity of the brain. Evidence reviewed systematically elsewhere (41) suggests that neurological outcome prediction in the acute stage of TBI cannot be done robustly if only one acute clinical descriptor of patient condition (e.g., the GCS-E) is used, although the joint use of several descriptors improves prediction accuracy. Thus, our study appears to confirm the advantage of using such multifaceted approaches to outcome prediction.

The substantial heterogeneity of TBI is an important reason for which our results should be interpreted with caution, and future replication of our results by studies involving larger cohorts would be welcome. Our power analysis indicates that the percentage of cortical regions associated with effect sizes below the required thresholds for Steps 1 and 2 of the analysis were 12 and 9%, respectively. Although none of the hypothesis tests associated with any of these latter regions were found to be significant, replication of our findings in a larger cohort would be very useful partly because (1) there is substantial variability in the statistical relationships explored here, (2) the effect sizes considered in this study should be estimated with greater confidence in a larger sample, and (3) the role of confounds associated with the administration of pharmacological agents (e.g., for sedation) is unclear. Nevertheless, the significance of each correlation between acute descriptors and chronic outcome measures—as reported explicitly in the Section “Results”—was tested in the context of adequate statistical power and are, thus, most trustworthy.

## Conclusion

The ability to forecast chronic atrophy patterns and extent following TBI based on clinical variables measured during the acute stage of this condition can aid in understanding the relationship between these variables and structural brain changes due to TBI. In this study, we illustrated how two readily recordable clinical descriptors (namely, GCS-E and the occurrence of epileptic seizures in the first week after TBI) can be correlated, with a high degree of statistical reliability, with the extent of cortical atrophy in brain regions which are involved in executive function, memory formation/retrieval, speech, and analytic processing. Accurate prognostication of functional degradation in the important domains of brain function affected by TBI may serve a useful role in formulating rehabilitation treatments. Very importantly, the amount of atrophy found in regions where important brain functions are localized has been found to be significantly correlated with the GOS-E; this suggests an important and novel correlative link between the acute presentation of TBI patients and their chronic functional outcome. In addition, this study helps to illustrate how acute clinical variables reflect structural atrophy subsequent to brain injury, as well as how such atrophy patterns can be related to the degradations of brain function observed in TBI. Future replication of our findings in larger cohorts is essential for more accurate interpretation and assessment of our findings.

## Ethics Statement

The study was designed and implemented in accordance with the Declaration of Helsinki, the U.S. Code of Federal Regulations (45 CFR 46), and with the approval of the Institutional Review Boards at the University of California, Los Angeles (UCLA), and the University of Southern California (USC). Signed informed consent was obtained from each patient or from their legally authorized representative prior to the study.

## Author Contributions

AI, PV, and JH conceived the project. PV recruited study volunteers and undertook data collection. AI, SG, AW, and KP analyzed the data. AI and JH interpreted the data. AI wrote the manuscript.

## Conflict of Interest Statement

The authors declare that the research was conducted in the absence of any commercial or financial relationships which could be construed as a potential conflict of interest. The reviewer DN and handling editor declared their shared affiliation.

## References

[1] Kraus MF Susmaras T Caughlin BP Walker CJ Sweeney JA Little DM. White matter integrity and cognition in chronic traumatic brain injury: a diffusion tensor imaging study. Brain (2007) 130:2508–19. 10.1093/brain/awm216 17872928

[2] Niogi SN Mukherjee P Ghajar J Johnson C Kolster RA Sarkar R Extent of microstructural white matter injury in postconcussive syndrome correlates with impaired cognitive reaction time: a 3T diffusion tensor imaging study of mild traumatic brain injury. AJNR Am J Neuroradiol (2008) 29:967–73. 10.3174/ajnr.A0970 18272556 PMC8128563

[3] Irimia A Van Horn JD. Epileptogenic focus localization in treatment-resistant post-traumatic epilepsy. J Clin Neurosci (2015) 22(4):627–31. 10.1016/j.jocn.2014.09.019 25542591 PMC4380645

[4] Sidaros A Engberg AW Sidaros K Liptrot MG Herning M Petersen P Diffusion tensor imaging during recovery from severe traumatic brain injury and relation to clinical outcome: a longitudinal study. Brain (2008) 131:559–72. 10.1093/brain/awm294 18083753

[5] Wilde EA Mccauley SR Hunter JV Bigler ED Chu Z Wang ZJ Diffusion tensor imaging of acute mild traumatic brain injury in adolescents. Neurology (2008) 70:948–55. 10.1212/01.wnl.0000305961.68029.54 18347317

[6] Sidaros A Skimminge A Liptrot MG Sidaros K Engberg AW Herning M Long-term global and regional brain volume changes following severe traumatic brain injury: a longitudinal study with clinical correlates. Neuroimage (2009) 44:1–8. 10.1016/j.neuroimage.2008.08.030 18804539

[7] Irimia A Chambers MC Torgerson CM Filippou M Hovda DA Alger JR Patient-tailored connectomics visualization for the assessment of white matter atrophy in traumatic brain injury. Front Neurol (2012) 3:10. 10.3389/fneur.2012.00010 22363313 PMC3275792

[8] Vespa PM Nuwer MR Nenov V Ronne-Engstrom E Hovda DA Bergsneider M Increased incidence and impact of nonconvulsive and convulsive seizures after traumatic brain injury as detected by continuous electroencephalographic monitoring. J Neurosurg (1999) 91:750–60. 10.3171/jns.1999.91.5.0750 10541231 PMC4347935

[9] Dale AM Fischl B Sereno MI Cortical surface-based analysis – I. Segmentation and surface reconstruction. Neuroimage (1999) 9:179–94. 10.1006/nimg.1998.0395 9931268

[10] Fischl B Sereno MI Dale AM Cortical surface-based analysis – II: inflation, flattening, and a surface-based coordinate system. Neuroimage (1999) 9:195–207. 10.1006/nimg.1998.0396 9931269

[11] Fischl B Salat DH Busa E Albert M Dieterich M Haselgrove C Whole brain segmentation: automated labeling of neuroanatomical structures in the human brain. Neuron (2002) 33:341–55. 10.1016/S0896-6273(02)00569-X 11832223

[12] Destrieux C Fischl B Dale A Halgren E. Automatic parcellation of human cortical gyri and sulci using standard anatomical nomenclature. Neuroimage (2010) 53:1–15. 10.1016/j.neuroimage.2010.06.010 20547229 PMC2937159

[13] Fischl B Salat DH Van Der Kouwe AJ Makris N Segonne F Quinn BT Sequence-independent segmentation of magnetic resonance images. Neuroimage (2004) 23(Suppl 1):S69–84. 10.1016/j.neuroimage.2004.07.016 15501102

[14] Rencher AC Methods of Multivariate Analysis. New York, NY: John Wiley & Sons, Inc (2002).

[15] Hochberg Y Benjamini Y More powerful procedures for multiple significance testing. Stat Med (1990) 9:811–8. 10.1002/sim.4780090710 2218183

[16] Cohen J Statistical Power Analysis for the Behavioral Sciences. Hillsdale, NJ: Lawrence Earlbaum Associates (1988).

[17] Gravel J D’angelo A Carriere B Crevier L Beauchamp MH Chauny JM Interventions provided in the acute phase for mild traumatic brain injury: a systematic review. Syst Rev (2013) 2:63. 10.1186/2046-4053-2-63 23924958 PMC3750385

[18] Merkley TL Bigler ED Wilde EA Mccauley SR Hunter JV Levin HS. Diffuse changes in cortical thickness in pediatric moderate-to-severe traumatic brain injury. J Neurotrauma (2008) 25:1343–5. 10.1089/neu.2008.0615 19061377 PMC2747789

[19] Wilde EA Merkley TL Bigler ED Max JE Schmidt AT Ayoub KW Longitudinal changes in cortical thickness in children after traumatic brain injury and their relation to behavioral regulation and emotional control. Int J Dev Neurosci (2012) 30:267–76. 10.1016/j.ijdevneu.2012.01.003 22266409 PMC3322311

[20] Wright MJ Mcarthur DL Alger JR Van Horn J Irimia A Filippou M Early metabolic crisis-related brain atrophy and cognition in traumatic brain injury. Brain Imaging Behav (2013) 7:307–15. 10.1007/s11682-013-9231-6 23636971 PMC4172457

[21] Wang X Xie H Cotton AS Tamburrino MB Brickman KR Lewis TJ Early cortical thickness change after mild traumatic brain injury following motor vehicle collision. J Neurotrauma (2015) 32:455–63. 10.1089/neu.2014.3492 25118568 PMC4376285

[22] Bigler ED. Traumatic brain injury, neuroimaging, and neurodegeneration. Front Hum Neurosci (2013) 7:395. 10.3389/fnhum.2013.00395 23964217 PMC3734373

[23] Mazzini L Cossa FM Angelino E Campini R Pastore I Monaco F. Posttraumatic epilepsy: neuroradiologic and neuropsychological assessment of long-term outcome. Epilepsia (2003) 44:569–74. 10.1046/j.1528-1157.2003.34902.x 12681007

[24] Mohanraj R Brodie MJ. Outcomes in newly diagnosed localization-related epilepsies. Seizure (2005) 14:318–23. 10.1016/j.seizure.2005.04.002 15876543

[25] Perucca P Dubeau F Gotman J. Intracranial electroencephalographic seizure-onset patterns: effect of underlying pathology. Brain (2014) 137:183–96. 10.1093/brain/awt299 24176980

[26] Braver TS Bongiolatti SR. The role of frontopolar cortex in subgoal processing during working memory. Neuroimage (2002) 15:523–36. 10.1006/nimg.2001.1019 11848695

[27] Petrides M Alivisatos B Meyer E Evans AC. Functional activation of the human frontal cortex during the performance of verbal working memory tasks. Proc Natl Acad Sci U S A (1993) 90:878–82. 10.1073/pnas.90.3.878 8430101 PMC45773

[28] Ridderinkhof KR Van Den Wildenberg WP Segalowitz SJ Carter CS. Neurocognitive mechanisms of cognitive control: the role of prefrontal cortex in action selection, response inhibition, performance monitoring, and reward-based learning. Brain Cogn (2004) 56:129–40. 10.1016/j.bandc.2004.09.016 15518930

[29] Moll J De Oliveira-Souza R Eslinger PJ Bramati IE Mourao-Miranda J Andreiuolo PA The neural correlates of moral sensitivity: a functional magnetic resonance imaging investigation of basic and moral emotions. J Neurosci (2002) 22:2730–6. 11923438 10.1523/JNEUROSCI.22-07-02730.2002 PMC6758288

[30] Leung HC Gore JC Goldman-Rakic PS. Sustained mnemonic response in the human middle frontal gyrus during on-line storage of spatial memoranda. J Cogn Neurosci (2002) 14:659–71. 10.1162/08989290260045882 12126506

[31] Aron AR Fletcher PC Bullmore ET Sahakian BJ Robbins TW Stop-signal inhibition disrupted by damage to right inferior frontal gyrus in humans. Nat Neurosci (2003) 6:115–6. 10.1038/nn1003 12536210

[32] Garavan H Ross TJ Stein EA. Right hemispheric dominance of inhibitory control: an event-related functional MRI study. Proc Natl Acad Sci U S A (1999) 96:8301–6. 10.1073/pnas.96.14.8301 10393989 PMC22229

[33] Binder JR Frost JA Hammeke TA Cox RW Rao SM Prieto T. Human brain language areas identified by functional magnetic resonance imaging. J Neurosci (1997) 17:353–62. 8987760 10.1523/JNEUROSCI.17-01-00353.1997 PMC6793702

[34] Puce A Allison T Asgari M Gore JC Mccarthy G. Differential sensitivity of human visual cortex to faces, letterstrings, and textures: a functional magnetic resonance imaging study. J Neurosci (1996) 16:5205–15. 8756449 10.1523/JNEUROSCI.16-16-05205.1996 PMC6579313

[35] Horwitz B Rumsey JM Donohue BC. Functional connectivity of the angular gyrus in normal reading and dyslexia. Proc Natl Acad Sci U S A (1998) 95:8939–44. 10.1073/pnas.95.15.8939 9671783 PMC21181

[36] Roux FE Boetto S Sacko O Chollet F Tremoulet M. Writing, calculating, and finger recognition in the region of the angular gyrus: a cortical stimulation study of Gerstmann syndrome. J Neurosurg (2003) 99:716–27. 10.3171/jns.2003.99.4.0716 14567608

[37] Kircher TT Senior C Phillips ML Benson PJ Bullmore ET Brammer M Towards a functional neuroanatomy of self processing: effects of faces and words. Brain Res Cogn Brain Res (2000) 10:133–44. 10.1016/S0926-6410(00)00036-7 10978701

[38] Corbetta M Miezin FM Dobmeyer S Shulman GL Petersen SE. Selective and divided attention during visual discriminations of shape, color, and speed: functional anatomy by positron emission tomography. J Neurosci (1991) 11:2383–402. 1869921 10.1523/JNEUROSCI.11-08-02383.1991 PMC6575512

[39] Menon V Rivera SM White CD Glover GH Reiss AL. Dissociating prefrontal and parietal cortex activation during arithmetic processing. Neuroimage (2000) 12:357–65. 10.1006/nimg.2000.0613 10988030

[40] Grabner RH Ansari D Koschutnig K Reishofer G Ebner F Neuper C. To retrieve or to calculate? Left angular gyrus mediates the retrieval of arithmetic facts during problem solving. Neuropsychologia (2009) 47:604–8. 10.1016/j.neuropsychologia.2008.10.013 19007800

[41] Mcnett M. A review of the predictive ability of Glasgow Coma Scale scores in head-injured patients. J Neurosci Nurs (2007) 39:68–75. 10.1097/01376517-200704000-00002 17477220

